# Mobility-Driven Design of PDMS-Modified Glassy Polymer Networks for Thermally Activated Shape Memory in Vat Photopolymerization

**DOI:** 10.3390/polym18131678

**Published:** 2026-07-07

**Authors:** Yura Choi, Namchul Cho

**Affiliations:** Department of Energy Engineering, Soonchunhyang University, Asan 31538, Republic of Korea; bnbn3238@sch.ac.kr

**Keywords:** 4D printing, glass transition modulation, polydimethylsiloxane, shape memory polymers, mobility–crosslink design

## Abstract

Glass-transition-driven shape memory polymers are promising materials for 4D printing because their thermally activated transition enables programmed deformation and recovery without relying on melting or crystallization-driven switching. In this study, PDMS-MMA-modified photocurable networks were designed for vat photopolymerization-based 4D printing by varying PDMS-MMA content and switching monomer structure while maintaining a fixed TMPTMA crosslinker content. The resin formulations were prepared using tert-butyl acrylate (tBA) or isobornyl acrylate (IBOA) as switching monomers, PDMS-MMA as a flexible mobility-regulating segment, and TMPTMA as a multifunctional crosslinker. The effects of formulation composition on printability, network formation, thermal stability, thermomechanical transition, mechanical properties, and shape memory behavior were systematically investigated. FT-IR analysis confirmed effective photocuring of the acrylate/methacrylate networks, while rheological evaluation showed that resin viscosity depended on monomer structure and PDMS-MMA content. DMA results revealed thermomechanical transition, although some formulations exhibited broad tan δ responses due to network heterogeneity and distributed segmental relaxation. Based on resin printability, printed-part resolution, and relatively well-defined tan δ transitions, T-15 and I-15 were selected as representative formulations for quantitative shape memory evaluation. Shape memory testing was conducted under force-control mode because stable strain-controlled programming was not achievable for the printed specimens. Both T-15 and I-15 exhibited high shape fixity over two programming–recovery cycles. I-15 showed stable recovery behavior with recovery ratios of 91.51% and 95.87%, whereas T-15 showed apparent over-recovery with recovery ratios exceeding 100%, likely due to residual stress release during reheating. Overall, these results demonstrate that thermally activated shape-memory performance is governed not only by the nominal transition temperature but also by the coupled effects of PDMS-mediated segmental mobility, switching monomer structure, mechanical integrity, and elastic energy storage within a fixed crosslinked network framework.

## 1. Introduction

Additive manufacturing (AM) has transformed the fabrication of complex and customized structures that are difficult to realize using conventional manufacturing techniques [1,2,3,4]. Among AM technologies, vat photopolymerization processes, including stereolithography (SLA) and digital light processing (DLP), are particularly attractive because of their high spatial resolution, smooth surface finish, and compatibility with a wide range of photocurable resins [5,6,7]. The incorporation of stimuli-responsive polymers into these processes has enabled four-dimensional (4D) printing, in which printed objects undergo programmed changes in shape or function over time in response to external stimuli such as heat, light, moisture, or electric fields [7,8,9,10].

Thermally responsive shape memory polymers (SMPs) are among the most widely investigated material systems for 4D printing because they combine low density, large programmable deformation, and tunable thermomechanical properties [10,11,12,13,14]. In a typical polymer network, the shape memory effect is governed by a permanent covalent network that defines the original shape and a reversible switching mechanism that fixes the temporary shape [1,15]. For glass-transition-based SMPs, deformation is introduced above *T_g_*, where polymer chains possess sufficient mobility, whereas cooling below *T_g_* immobilizes the temporary shape through vitrification [16,17]. Subsequent reheating above *T_g_* restores chain mobility and releases stored elastic energy, thereby driving shape recovery [1,2,3,18].

Although *T_g_* is often used as the primary design parameter for thermally triggered SMPs, transition temperature alone does not fully determine actuation performance [19]. Shape recovery efficiency, reversible strain, recovery stress, and cyclic stability are collectively governed by segmental mobility, crosslink density, elastic energy storage, and relaxation kinetics within the polymer network [10,19,20]. Consequently, systems designed solely by shifting *T_g_* can suffer from limited reversible deformation, brittle mechanical behavior, or narrow actuation windows [18].

These limitations are particularly important for vat-photopolymerizable 4D printing resins. Acrylate and epoxy-based photocurable networks are commonly used because they provide rapid curing, high printing fidelity, and structural stability [8,12,21]. However, their highly crosslinked architectures often restrict chain mobility and reduce extensibility. Increasing crosslink density can enhance recovery stress and shape stability, but it also suppresses chain motion and limits reversible strain [7,10,12]. Conversely, reducing crosslink density improves deformability but can weaken elastic energy storage and recovery force [19,20]. This mobility–stiffness trade-off remains a central challenge in the design of glass-transition-based 4D printable materials.

Incorporating flexible silicone segments such as polydimethylsiloxane (PDMS) provides a promising strategy for regulating molecular mobility in crosslinked polymer networks [22,23,24,25]. Recent studies on silicone- or PDMS-containing photocurable resins further suggest that silicone segments can be incorporated into vat photopolymerization compatible SMP networks to tailor flexibility, thermomechanical response, and shape memory behavior [26]. Owing to its flexible Si–O backbone, low intrinsic *T_g_*, and elastic recovery characteristics, PDMS can act as a soft segment that lowers cooperative relaxation temperatures and improves deformability in glassy polymer matrices [22,24,27]. However, excessive PDMS incorporation may dilute network connectivity, reduce stored elastic energy, and compromise recovery force or dimensional stability [22,28,29]. Therefore, efficient glass-transition-based 4D printing resins require a formulation strategy that balances segmental mobility with network constraint rather than simply shifting *T_g_* [29].

Despite increasing research on *T_g_*-modulated SMP systems, a generalized framework that explicitly correlates mobility regulation with actuation performance in SLA-printable networks remains insufficiently established. Previous studies on (meth)acrylate-based SMP networks have shown that thermomechanical properties and shape recovery behavior are strongly affected by monomer structure, crosslink density, toughness, and rubbery modulus, rather than *T_g_* alone [1,21]. However, most prior studies have focused on tuning transition temperature or demonstrating printable SMP behavior, while fewer have explicitly examined how PDMS-mediated mobility regulation and switching monomer structure jointly affect thermally activated actuation under a fixed crosslinked network framework [30].

In this study, we introduce a mobility-driven design framework for thermoresponsive 4D printable resins by incorporating PDMS soft segments into photocrosslinkable acrylate/methacrylate networks. The design strategy focuses on two key formulation variables: segmental mobility, regulated by PDMS-MMA incorporation, and switching monomer structure, controlled by comparing tert-butyl acrylate and isobornyl acrylate. TMPTMA was maintained at a constant concentration to provide a fixed multifunctional crosslinked network framework. By systematically varying PDMS-MMA content while comparing tBA- and IBOA-based formulations, we establish a structure property actuation relationship for thermomechanical transition activated shape memory behavior. Overall, this work moves beyond conventional *T_g_*-centered design by showing that thermal actuation performance is governed by the coupled balance among segmental mobility, switching monomer structure, mechanical integrity, and elastic energy storage.

## 2. Materials and Methods

### 2.1. Materials

Triethylene glycol dimethacrylate (TEGDMA) was used as the primary dimethacrylate monomer forming the glassy polymer backbone of the photocurable network. tert-Butyl acrylate (tBA) and isobornyl acrylate (IBOA) were employed as mono-acrylate switching monomers to regulate the glass transition behavior and thermomechanical properties of the network.

**Figure 1:**
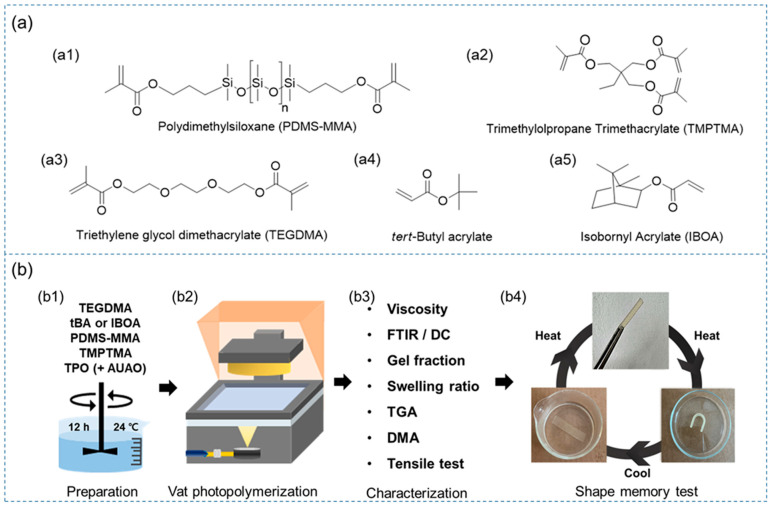
Chemical structures and experimental workflow of PDMS-MMA-modified photocurable networks for vat photopolymerization-based thermally activated shape memory. (**a**) Representative chemical structures of the resin components: (**a1**) methacrylate-functionalized polydimethylsiloxane (PDMS-MMA), (**a2**) trimethylolpropane trimethacrylate (TMPTMA), (**a3**) triethylene glycol dimethacrylate (TEGDMA), (**a4**) tert-butyl acrylate (tBA), and (**a5**) isobornyl acrylate (IBOA). (**b**) Experimental workflow: (**b1**) resin preparation by mixing TEGDMA, tBA or IBOA, PDMS-MMA, TMPTMA, TPO, and AUAO for 12 h at 24 °C; (**b2**) vat photopolymerization; (**b3**) characterization by viscosity measurement, FTIR/degree of conversion (DC), gel fraction, swelling ratio, TGA, DMA, and tensile testing; and (**b4**) thermally induced shape-memory testing.

Methacrylate-functionalized polydimethylsiloxane (PDMS-MMA) was incorporated as a flexible soft segment to regulate segmental mobility within the network. Trimethylolpropane trimethacrylate (TMPTMA) was used as a multifunctional crosslinker to control network crosslink density and elastic energy storage capacity. Diphenyl(2,4,6-trimethylbenzoyl)phosphine oxide (TPO) was used as a Type-I photoinitiator for free-radical photopolymerization under 405 nm UV irradiation (Figure 1 and Table 1).

TMPTMA and TPO were obtained from Miwon Chemical Co., Ltd. (Yongin, Republic of Korea). PDMS-MMA was purchased from Burkeglimanco Co., Ltd. (Asan, Republic of Korea). TEGDMA, tert-butyl acrylate, and isobornyl acrylate were purchased from BLD Pharm Co., Ltd. (Shanghai, China). The bi-functional aliphatic urethane acrylate oligomer was obtained from Miwon Chemical Co., Ltd. (Yongin, Republic of Korea).

### 2.2. Resin Formulation

The compositions of the photocurable resins used in this study are summarized in Table 2. To investigate the effects of segmental mobility and switching monomer structure, two formulation series were prepared. In the tBA-based series, tert-butyl acrylate was used as the switching monomer, while the PDMS-MMA content was varied from 0 to 15 wt%. In the IBOA-based series, isobornyl acrylate was used as the switching monomer under the same PDMS-MMA variation. In all formulations, TMPTMA was fixed at 10 wt% to provide a constant multifunctional crosslinked network framework. All components were weighed according to the compositions listed in Table 2 and mixed in amber glass vials. The mixtures were magnetically stirred for 12 h at room temperature to obtain homogeneous photocurable resins.

### 2.3. Characterization Methods

#### 2.3.1. Fourier Transform Infrared Spectroscopy (FTIR)

Fourier transform infrared spectroscopy (FTIR) was performed to confirm the chemical structure of the photocured networks and to evaluate the conversion of acrylate double bonds. FT-IR spectra were recorded using an FT-IR spectrometer (FT/IR-4600, JASCO Corporation, Tokyo, Japan) in attenuated total reflectance (ATR) mode. Spectra were collected in the range of 3500–500 cm^−1^ with a resolution of 4 cm^−1^ and 32 scans per sample.

The degree of conversion (DC) was calculated from the decrease in the acrylate C=C absorbance band at approximately 1635–1640 cm^−1^ before and after UV curing. The spectra were baseline-corrected before comparing the C=C peak intensity.

(1) $$DC\left(\%\right)=\left(1-\frac{A_{C=C,\;\;cured}}{A_{C=C,\;\;uncured}}\right)\times 100\;$$

where *A_C_*_=*C*,cured_ and *A_C_*_=*C*,uncured_ are the baseline-corrected absorbance intensities of the acrylate C=C band after and before UV curing, respectively.

#### 2.3.2. Gel Fraction and Swelling Measurements

Gel content was determined via Soxhlet extraction using tetrahydrofuran (THF) for 24 h. After drying at 60 °C under vacuum, gel fraction was calculated [5].

(2) $$\mathrm{Gel}\mathrm{Fraction}\;(\%)=m_{2}/m_{1}\times 100$$

where *m*_1_ is initial mass and *m*_2_ is dried extracted mass.

Network stability and crosslink density were evaluated through swelling experiments. Printed samples were immersed in THF at 37 °C for up to 10 days. At predetermined time intervals, the samples were removed, gently dried, and weighed. The swelling ratio (SR) was calculated using:

(3) $$swelling\;ratio=\frac{W_{s\;}-\;W_{d}\;}{\;W_{d}}\;\times 100$$

where *W_s_* is swollen sample mass and *W_d_* is dried sample mass.

#### 2.3.3. Thermogravimetric Analysis (TGA)

The thermal stability of the cured PDMS-MMA-modified networks was evaluated using thermogravimetric analysis (TGA). TGA was performed using a thermogravimetric analyzer (Q50, TA Instruments, New Castle, DE, USA), in which approximately 10–20 mg of each cured sample was heated from room temperature to 500 °C at a heating rate of 10 °C min^−1^ under a nitrogen atmosphere.

#### 2.3.4. Dynamic Mechanical Analysis (DMA)

Dynamic mechanical properties were evaluated using a dynamic mechanical analyzer (DMA Q800, TA Instruments) in tensile mode. Rectangular specimens (dimensions: 20 mm × 5 mm × 1 mm) were tested at a frequency of 1 Hz with a temperature ramp from −50 °C to 120 °C at 3 °C min^−1^. The storage modulus (*E′*), loss modulus (*E″*), and damping factor (tan δ) were recorded to evaluate the thermomechanical properties and segmental mobility of the photocured networks. The tan δ peak temperature was used as the representative DMA-derived thermomechanical transition temperature for comparing the photocured networks.

#### 2.3.5. Mechanical Testing

Uniaxial tensile tests were performed using a universal testing machine (Instron 5943, Instron Corporation, Norwood, MA, USA). Dog-bone-shaped specimens were prepared according to ASTM D638 specifications. The tests were conducted at room temperature with a crosshead speed of 5 mm min^−1^. At least three specimens were tested for each formulation, and the average values were reported.

#### 2.3.6. Shape Memory Actuation Tests

Thermally induced one-way shape memory behavior was evaluated for the selected T-15 and I-15 formulations using rectangular strip specimens under force-control mode. Force-control mode was selected because stable strain-controlled programming was not achievable for the printed specimens during preliminary testing. The specimens were first heated to the programming temperature and equilibrated before mechanical deformation. A predefined force was then gradually applied to deform the specimen. After reaching the target force or target deformation, the applied force was maintained while the specimen was cooled to room temperature to fix the temporary shape.

After cooling, the external force was removed, and the fixed strain was recorded. Shape recovery was triggered by reheating the specimen to the recovery temperature under an unloaded condition. The strain response during programming, fixing, and recovery was recorded throughout the test. Although the programming step was conducted under force-control mode, the recorded strain values were used to quantify the shape memory performance.

The programming and recovery temperature was set to 180–185 °C to ensure sufficient chain mobility and stable deformation/recovery during the force-controlled shape memory test. Although DMA indicated a tan δ transition around 90–100 °C, reliable shape memory deformation could not be obtained at this temperature range due to insufficient specimen compliance and instrumental force limitations during preliminary testing.

The shape fixity ratio (*R_f_*) and shape recovery ratio (*R_r_*) were calculated from the measured strain values using the following equations [17,31]:

(4) $$R_{f}=\frac{\varepsilon_{fix}(N)\;}{\;\varepsilon_{load}(N)}\;\times 100$$

(5) $$R_{r}=\frac{\varepsilon_{fix}\left(N\right)-\varepsilon_{rec}(N)\;}{\varepsilon_{fix}\left(N\right)-\varepsilon_{rec}(N-1)}\times 100$$

In addition to the quantitative force-controlled shape memory cycling, a qualitative visual demonstration was conducted to confirm macroscopic shape recovery under a milder thermal condition. For this demonstration, the printed T-15 strip was heated to 110 °C, slightly above the thermomechanical transition region identified by DMA, manually deformed, and cooled to 15–20 °C under constraint to fix the temporary shape. After removal of the constraint, shape recovery was triggered by reheating the specimen to 110 °C. The recovered configuration was then observed after cooling back to room temperature. This visual demonstration was performed separately from the quantitative force-controlled test, which required a higher programming/recovery temperature of 180–185 °C because stable gripping and deformation could not be achieved near the DMA-derived *T_g_* range.

## 3. Results and Discussion

### 3.1. Photocuring Behavior and Network Formation

**Figure 2:**
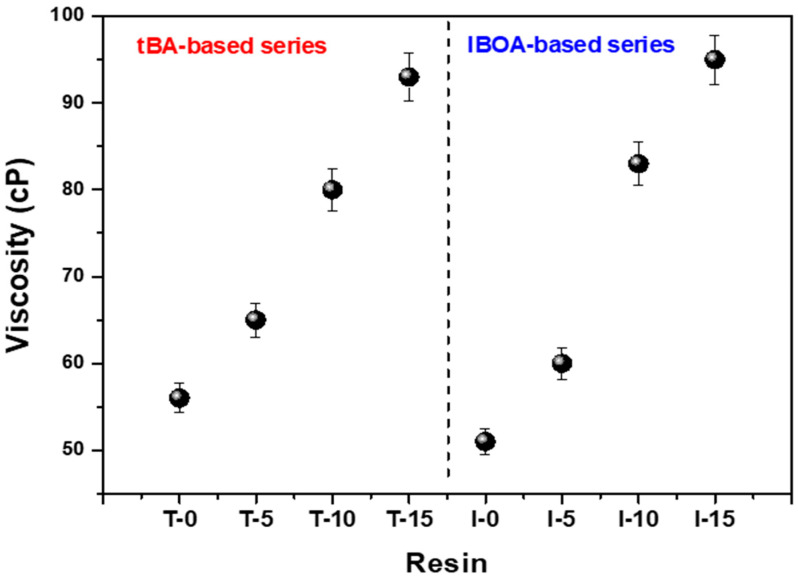
Viscosity of tBA- and IBOA-based photocurable resin formulations with different PDMS-MMA contents. T-series and I-series indicate tBA- and IBOA-based formulations, respectively, and the numbers in the sample names indicate the PDMS-MMA content in wt%.

Before photocuring, the viscosity of the resin formulations was evaluated to assess their suitability for vat photopolymerization. As shown in Figure 2, resin viscosity increased with increasing PDMS-MMA content in both the tBA- and IBOA-based series, indicating that the incorporation of PDMS-MMA affected flow behavior prior to printing. Nevertheless, all formulations remained within a relatively low-viscosity range, supporting their processability as photocurable resins for vat photopolymerization.

**Figure 3:**
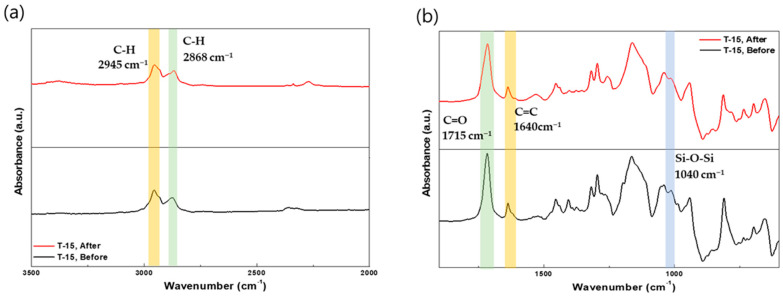
FT-IR absorbance spectra of the T-15 formulation before and after UV curing. Enlarged spectra are shown in the regions of (**a**) 3500–2000 cm^−1^ and (**b**) 1900–600 cm^−1^. The characteristic bands assigned to C–H stretching, C=O stretching, acrylate C=C stretching, and Si–O–Si vibration are labeled.

To verify successful photocuring and evaluate how formulation variables affect network formation, Fourier transform infrared (FT-IR) spectroscopy, degree of conversion (DC) analysis, and solvent-based network characterization were performed. As shown in Figure 3, the FT-IR spectra are presented as absorbance spectra. After UV curing, the absorbance band assigned to acrylate C=C stretching at approximately 1625–1650 cm^−1^ markedly decreased after UV curing, indicating effective consumption of reactive double bonds during photopolymerization. In the FT-IR spectra, the reduction of the C=C band was clearly observed while the carbonyl (C=O) band near 1690–1735 cm^−1^ remained evident, confirming that the acrylate groups were polymerized without decomposition of the ester-containing backbone. In addition, the Si–O–Si absorption band around 1040 cm^−1^ remained present in PDMS-containing samples, demonstrating that the PDMS-MMA segment was successfully incorporated into the cured network and remained chemically stable after irradiation.

The quantitative FT-IR analysis further showed that all formulations achieved relatively high conversion values despite compositional variation. According to Table 3, increasing PDMS-MMA content led to a slight but systematic decrease in DC in both resin series. In the tBA-based system, DC decreased from 92 ± 2% for T-0 to 88 ± 3% for T-15, while in the IBOA-based system it decreased from 91 ± 2% for I-0 to 87 ± 3% for I-15. This trend suggests that the incorporation of flexible PDMS segments slightly lowers the effective concentration of polymerizable acrylate groups and increases local chain mobility during curing, which may reduce radical propagation efficiency to a limited extent. Nevertheless, all samples maintained DC values above approximately 85%**,** indicating that PDMS incorporation did not critically hinder photocuring and that sufficient covalent network formation was achieved for subsequent thermomechanical evaluation.

**Figure 4:**
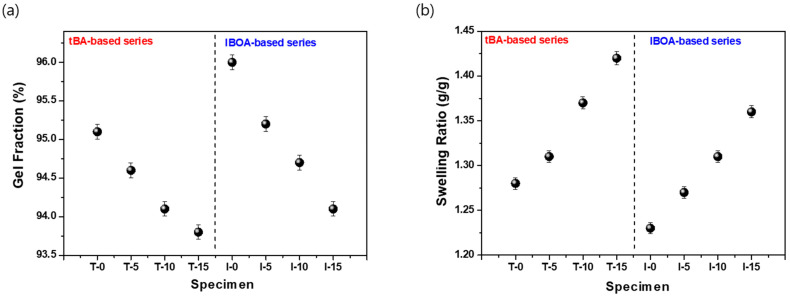
Gel fraction and swelling ratio of photocured tBA- and IBOA-based networks with varying PDMS-MMA contents. (**a**) Gel fraction as a function of PDMS-MMA content for the tBA- and IBOA-based series. (**b**) Swelling ratio as a function of PDMS-MMA content for the tBA- and IBOA-based series. The left and right groups in each graph correspond to the tBA-based and IBOA-based series, respectively.

The network structure was further examined by swelling and gel fraction measurements, and the results are presented in Figure 4a,b. In both the tBA- and IBOA-based systems, the swelling ratio generally increased with increasing PDMS-MMA content, whereas the gel fraction showed a slight decreasing tendency. This result indicates that incorporation of PDMS soft segments relaxed the network structure and increased solvent uptake, which is consistent with reduced packing efficiency and enhanced segmental flexibility. At the same time, the gel fraction remained high across all formulations, demonstrating that the networks still possessed sufficient connectivity and insoluble fraction even after mobility enhancement through PDMS addition. Thus, PDMS-MMA appears to act not as a network disruptor, but as a mobility-regulating soft segment that loosens the network without destroying its overall integrity. The differences between the tBA- and IBOA-based systems can be attributed to the molecular structure of the switching monomers. The relatively flexible tBA-containing networks and the bulkier IBOA-containing networks may differ in chain packing, local free volume, and solvent accessibility, leading to formulation dependent swelling and gel fraction behavior. Taken together, the results from Figure 3, Table 3, and Figure 4 demonstrate that all formulations formed well-developed photocured networks with high conversion, stable PDMS incorporation, and composition-dependent network structures. More specifically, increasing PDMS-MMA content slightly reduced curing efficiency and increased swelling due to enhanced flexibility, while the switching monomer structure influenced the compactness and relaxation behavior of the glassy polymer networks.

### 3.2. Thermal Stability of PDMS-Modified Networks

#### 3.2.1. Thermal Stability by TGA

**Figure 5:**
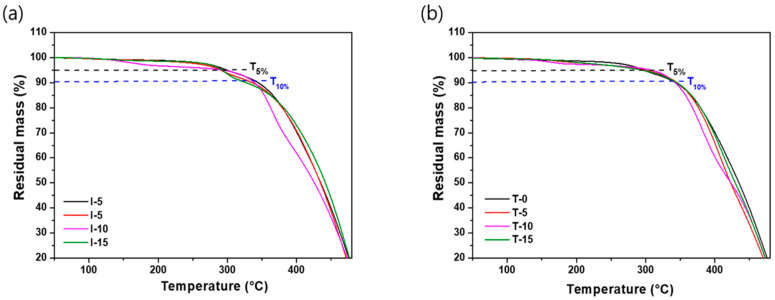
TGA thermograms of photocured networks with varying PDMS-MMA contents. (**a**) IBOA-based series (I-0, I-5, I-10, and I-15). (**b**) tBA-based series (T-0, T-5, T-10, and T-15). The y-axis represents residual mass (%), and the dashed horizontal lines indicate the residual mass levels used to determine T_5%_ and T_10%_. Characteristic thermal stability parameters are summarized in Table 4.

Thermal stability of the photocured networks was evaluated by thermogravimetric analysis (TGA), and the corresponding weight-loss curves are presented in Table 4 and Figure 5. All formulations exhibited relatively similar decomposition profiles, indicating that the incorporation of PDMS and the variation of switching monomer structure did not significantly compromise the overall thermal stability of the photocured networks within the investigated composition range. In all cases, negligible mass loss was observed in the low-temperature region, suggesting that the cured samples contained minimal volatile residues and that the photocuring process produced structurally stable crosslinked networks.

As shown in Figure 5, the major thermal degradation of all samples occurred at elevated temperatures, with the main weight-loss step appearing in the approximate range of 300–450 °C. This behavior is characteristic of highly crosslinked acrylate-based polymer networks, in which decomposition is associated with scission of the polymer backbone and breakdown of the crosslinked structure. The relatively close overlap of the TGA curves suggests that the primary degradation mechanism remained broadly similar across both the tBA and IBOA-based systems, despite the compositional differences introduced by PDMS content.

A closer comparison of the curves indicates that PDMS incorporation may slightly influence the onset and progression of thermal degradation, but the effect appears modest rather than drastic. This result suggests that PDMS mainly functions as a mobility-regulating soft segment under the present formulation conditions, while maintaining the bulk thermal robustness required for handling and thermally triggered actuation. In other words, the introduction of PDMS improves network flexibility without causing a substantial loss in thermal resistance at the application-relevant level. In addition, the tBA- and IBOA-based formulations showed comparable overall thermal decomposition behavior, implying that the switching monomer structure had a stronger influence on thermomechanical transition behavior than on gross thermal stability. This is an important distinction for the present study, because it indicates that changes in actuation behavior are more likely to originate from differences in segmental mobility and network constraint rather than from insufficient thermal durability of the cured materials [2].

Overall, the TGA results confirm that all photocured formulations possess adequate thermal stability for subsequent thermomechanical characterization and shape memory evaluation. Therefore, within the studied composition range, PDMS incorporation and switching monomer variation can be used to tailor network mobility without severely compromising the thermal integrity of the printed networks.

#### 3.2.2. Thermomechanical Transition by DMA

**Figure 6:**
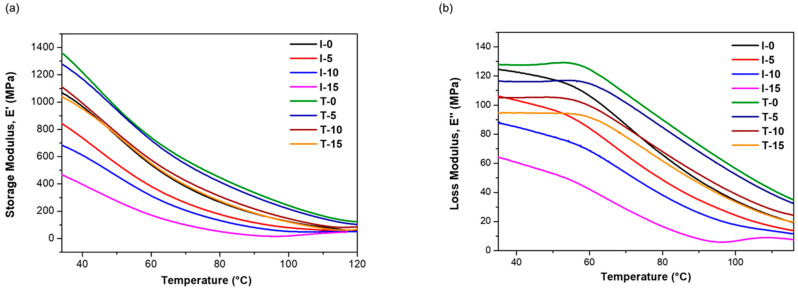

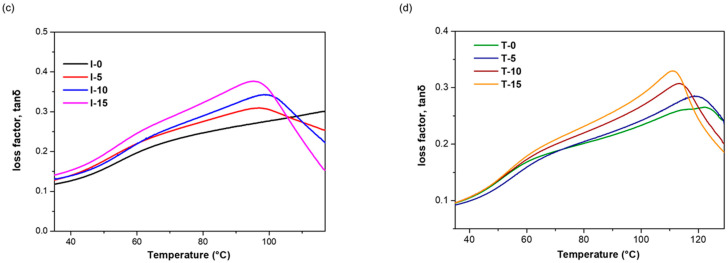
DMA results of photocured tBA- and IBOA-based networks: (**a**) storage modulus *E*′, (**b**) loss modulus *E*″, (**c**) tan δ curves of IBOA-based formulations, and (**d**) tan δ curves of tBA-based formulations.

Dynamic mechanical analysis was performed to evaluate the thermomechanical transition and temperature-dependent segmental mobility of the photocured networks. As shown in Figure 6, all formulations exhibited a decrease in storage modulus with increasing temperature, indicating thermally activated softening from the glassy state to a more mobile state. This modulus transition is essential for thermally activated shape-memory behavior because the printed networks must be sufficiently rigid below *T_g_* for shape fixation while becoming deformable above the transition region for programming and recovery. The DMA-derived thermomechanical transition parameters, including the *E″* transition/inflection temperature, *E″* at the transition point, tan δ peak temperature, and tan δmax, are summarized in Table 5.

Preliminary DSC measurements were also performed for the neat resin components and selected photocured networks. However, the photocured networks did not show clearly resolved calorimetric *T_g_* transitions within the accessible measurement range. This may be attributed to the highly crosslinked and multi-component nature of the photocured acrylate/methacrylate networks, which can produce broad and distributed relaxation behavior rather than a sharp DSC transition. In addition, Transition-related information from the neat uncured components cannot be directly assigned to the final photocured networks after photopolymerization. Therefore, DMA was used as the primary method for evaluating the thermomechanical transition of the cured networks, and the tan δ peak temperature was used as a representative transition parameter for comparing the formulations.

The tan δ curves showed that the transition behavior strongly depended on formulation composition. Some formulations exhibited broad and less-defined tan δ responses, suggesting distributed relaxation behavior caused by network heterogeneity, PDMS-MMA incorporation, and differences in local crosslink density. Such broad transitions are commonly observed in crosslinked photopolymer networks, where segmental relaxation occurs over a wide temperature range rather than at a single sharply defined *T_g_* [18]. In contrast, T-15 and I-15 showed relatively clearer tan δ peaks among the investigated formulations, indicating a more identifiable *T_g_*-related switching region for thermomechanical programming.

The difference between the tBA- and IBOA-based systems can be attributed to the different molecular structures of the switching monomers. The tBA-based network provides relatively flexible acrylate segments, whereas the IBOA-based network contains a bulky bicyclic structure that can influence chain packing, damping behavior, and glass transition broadness. Overall, the DMA results indicate that the thermally activated actuation behavior of the present networks is governed not only by the nominal transition temperature but also by the breadth of the relaxation transition, PDMS-MMA-induced mobility, and network constraint.

### 3.3. Tensile Mechanical Properties

**Figure 7:**
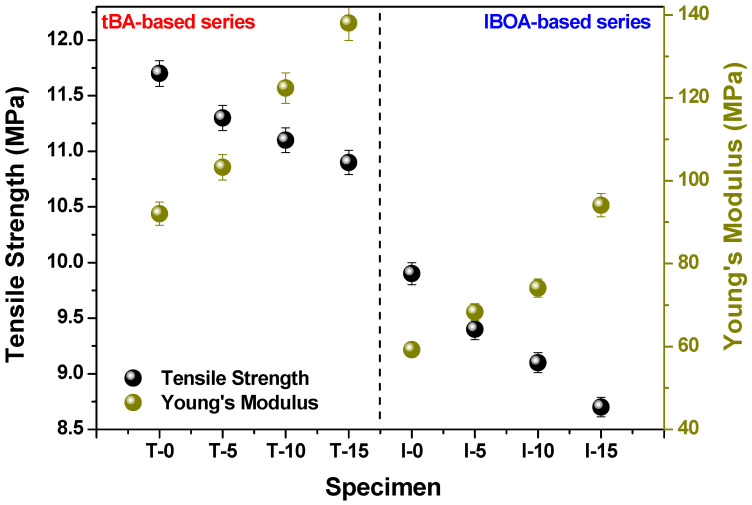
Tensile strength and Young’s modulus of photocured tBA- and IBOA-based networks with varying PDMS-MMA contents. The left and right groups correspond to the tBA-based and IBOA-based series, respectively (25 ± 1 °C).

The mechanical properties of the photocured networks are summarized in Table 6 and Figure 7. In both the tBA- and IBOA-based systems, increasing PDMS-MMA content led to a slight decrease in tensile strength, while Young’s modulus increased. For the tBA-based formulations, tensile strength decreased from 11.7 MPa for T-0 to 10.9 MPa for T-15, whereas Young’s modulus increased from 92.05 MPa to 138.04 MPa. Similarly, the IBOA-based formulations showed a decrease in tensile strength from 9.9 MPa for I-0 to 8.7 MPa for I-15, while Young’s modulus increased from 59.25 MPa to 94.10 MPa. These results suggest that PDMS-MMA incorporation increased network stiffness while slightly reducing ultimate tensile strength, possibly due to changes in network packing, phase compatibility, or stress transfer within the photocured acrylate/methacrylate network.

### 3.4. Shape Memory Performance

**Figure 8:**
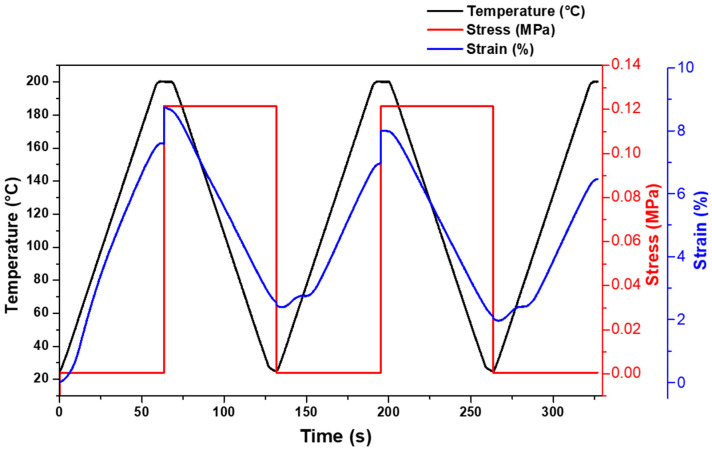
Force-controlled thermomechanical shape-memory cycling of the T-15 formulation over two programming–recovery cycles. The black, red, and blue curves correspond to temperature (°C), stress (MPa), and strain (%), respectively.

The shape memory test was performed at 180–185 °C rather than at the DMA-derived transition region of 90–100 °C because stable deformation and recovery were difficult to obtain at 90–100 °C under the available testing conditions. The higher programming/recovery temperature provided sufficient chain mobility for reliable force-controlled programming and unloaded recovery. The one-way shape memory performance of representative T-15 and I-15 formulations was evaluated over two thermomechanical cycles, as summarized in Table 7 and Figure 8. Before quantitative shape memory evaluation, representative formulations were selected based on printability, dimensional resolution, and thermomechanical transition behavior. Because the present resin system was designed for vat photopolymerization-based 3D printing, low viscosity and sufficient printing resolution were first considered as practical selection criteria. In addition, since the shape memory response of this system is associated with the tan δ relaxation behavior the clarity of the tan δ relaxation peak in DMA was also considered. Some formulations exhibited broad and less-defined tan δ transitions, which made it difficult to clearly define the effective switching region. Among the investigated formulations, T-15 and I-15 showed relatively clearer tan δ peaks while maintaining acceptable printability and printed-part resolution. Therefore, T-15 and I-15 were selected as representative formulations for quantitative one-way shape memory evaluation. Because the shape memory test was conducted under force-control mode, stable specimen gripping and dimensional integrity during loading, cooling, and recovery were also essential for reliable quantification.

Both formulations exhibited high shape fixity, indicating that the temporary shape could be effectively fixed after cooling below the glass transition region. The I-15 sample showed Rf values of 95.32% and 96.18% during the first and second cycles, respectively. The T-15 sample exhibited slightly higher Rf values of 98.27% and 98.32%, confirming excellent temporary-shape locking capability. These results suggest that the photocured networks retained sufficient rigidity in the cooled state despite the incorporation of flexible PDMS-MMA segments.

The recovery behavior differed between the two formulations. I-15 exhibited recovery ratios of 91.51% and 95.87% over two cycles, indicating stable and efficient shape recovery. The improved recovery in the second cycle suggests that the network became more stable after the initial thermomechanical cycle, likely due to stress relaxation and more uniform chain rearrangement.

For T-15, the calculated recovery ratios exceeded 100%, reaching 125.29% in the first cycle and 118.40% in the second cycle. This apparent recovery ratio above 100% is attributed to over-recovery, as reflected by the negative residual strain values after recovery. Such over-recovery may originate from residual stress release or slight reverse deformation during reheating. Therefore, T-15 demonstrates strong recovery capability, although the over-recovery behavior should be considered when evaluating dimensional accuracy and actuation controllability.

Overall, the shape memory results confirm that the selected PDMS-MMA-modified photocured networks can achieve high shape fixity and efficient thermal recovery. The I-15 formulation showed stable recovery close to complete recovery, whereas the T-15 formulation exhibited stronger recovery accompanied by over-recovery. These results support the proposed mobility–crosslink design concept, in which PDMS-MMA enhances segmental mobility while the crosslinked acrylate network stores sufficient elastic energy for shape recovery.

### 3.5. Structure–Property–Actuation Relationship

**Figure 9:**
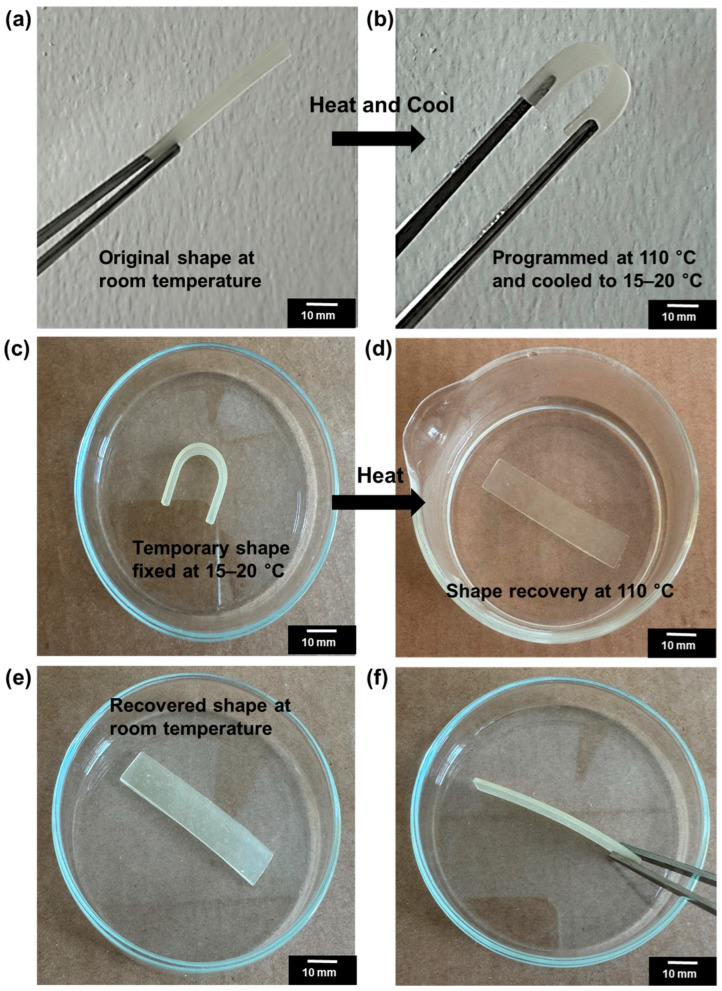
Qualitative visual demonstration of the thermally induced one-way shape memory behavior of the T-15 formulation. (**a**) Original straight shape of the printed strip at room temperature. (**b**) Programming of the specimen by heating to 110 °C, slightly above the DMA-derived transition region, followed by mechanical deformation and cooling to 15–20 °C under constraint. (**c**) Temporary U-shaped configuration fixed after cooling to 15–20 °C. (**d**) Shape recovery triggered by reheating the fixed specimen to 110 °C. (**e**) Recovered straight configuration after reheating and subsequent cooling to room temperature, shown from the top view. (**f**) Side view of the recovered specimen.

Figure 9 presents a qualitative visual demonstration of the macroscopic one-way shape memory behavior of the T-15 formulation. Unlike the quantitative force-controlled cycling test, which was conducted at 180–185 °C because stable gripping and deformation were not achievable near the DMA-derived *T_g_* range, the visual demonstration was performed at 110 °C to confirm macroscopic thermally induced recovery under a milder heating condition. The printed strip was first heated to 110 °C, mechanically deformed, and cooled to 15–20 °C under constraint to fix a temporary U-shaped configuration. Upon reheating to 110 °C, the specimen recovered toward its original straight shape, and the recovered configuration was retained after cooling to room temperature. The recovered specimens shown in Figure 9e,f correspond to the same recovery state observed from different orientations.

The mechanical results showed that increasing PDMS-MMA content increased Young’s modulus while slightly reducing tensile strength, indicating that PDMS-MMA incorporation modified the stiffness–strength balance of the photocured networks. Despite the slight decrease in tensile strength, the high-PDMS formulations retained sufficient mechanical integrity for shape-memory programming and recovery. In particular, I-15 showed stable recovery close to complete recovery, whereas T-15 showed apparent over-recovery, indicating stronger stored elastic recovery accompanied by residual stress release.

The DMA-derived transition parameters further indicate that the transition temperature was influenced by both PDMS-MMA content and switching monomer structure. The incorporation of PDMS-MMA introduced flexible siloxane segments into the photocured networks, which enhanced segmental mobility and broadened the relaxation transition. In contrast, the bulky cyclic structure of IBOA restricted local chain mobility and affected chain packing, stiffness, and the position of the transition temperature. Therefore, the observed shape-memory behavior should be interpreted not only from the nominal tan δ peak temperature but also from the transition breadth, rubbery-state mobility, network constraint, and stored elastic energy.

These results indicate that efficient thermally activated shape-memory behavior is not governed by *T_g_* alone. Instead, actuation performance depends on the coupled control of glass transition behavior, segmental mobility, network constraint, and elastic energy storage. Therefore, the present formulation strategy provides a useful design guideline for vat-photopolymerization-printable thermoresponsive 4D resins.

The difference between the DMA-derived thermomechanical transition region and the practical shape memory testing temperature suggests that nominal *T_g_* alone is insufficient to define the actuation condition of the printed networks [16]. Although tan δ peaks appeared around 90–100 °C, stable force-controlled programming and recovery required a higher temperature of 180–185 °C, where the network possessed sufficient chain mobility under the available testing conditions. Therefore, the actuation behavior should be interpreted through the combined effects of *T_g_*-related relaxation, segmental mobility, mechanical constraint, and testing conditions.

To further position the present PDMS-MMA-modified photocurable networks relative to previously reported shape-memory polymer systems, representative vat-photopolymerization-based and PDMS/silicone-containing SMP systems are compared in Table 8. Because shape-memory performance is strongly affected by resin chemistry, programming temperature, deformation mode, applied strain or stress, and cycle number, the comparison is intended to provide qualitative positioning rather than a direct one-to-one quantitative ranking.

As summarized in Table 8, the present I-15 formulation exhibited high shape fixity and stable recovery behavior comparable to representative vat-photopolymerization-based SMP systems. In contrast, the T-15 formulation showed apparent over-recovery, suggesting strong stored elastic recovery accompanied by residual stress release. Compared with previously reported systems, the present formulation strategy is distinguished by the use of PDMS-MMA as a mobility-regulating soft segment within a fixed TMPTMA-crosslinked acrylate/methacrylate network. These results highlight that actuation performance is governed not only by the nominal thermomechanical transition but also by the balance among segmental mobility, network constraint, and elastic energy storage.

## 4. Conclusions

In this study, PDMS-MMA-modified glassy polymer networks were designed as *T_g_*-related thermally activated shape memory materials for vat photopolymerization-based 4D printing. By varying PDMS-MMA content and comparing tBA- and IBOA-based formulations under a fixed TMPTMA crosslinker content, the effects of segmental mobility and switching monomer structure on thermomechanical and shape memory behavior were investigated. FT-IR analysis confirmed effective photocuring of the acrylate/methacrylate networks, while TGA demonstrated that all formulations maintained sufficient thermal stability for thermally triggered actuation. DMA results showed temperature-dependent softening behavior, supporting the thermally activated actuation mechanism of the printed networks. Mechanical testing revealed that increasing PDMS-MMA content increased Young’s modulus while slightly reducing tensile strength, suggesting that PDMS-MMA incorporation modified the stiffness–strength balance of the photocured networks without severely compromising structural integrity. Representative I-15 and T-15 formulations exhibited high shape fixity over two programming–recovery cycles. I-15 showed stable recovery behavior with recovery ratios of 91.51% and 95.87%, whereas T-15 exhibited apparent over-recovery with recovery ratios above 100%, likely due to residual stress release during reheating. These results demonstrate that the balance between PDMS-induced segmental mobility and network constraint plays a critical role in determining thermally activated shape-memory performance.

Overall, this work establishes a mobility–structure design strategy for vat-photopolymerization-printable shape-memory resins. The main contribution of this study is the demonstration that thermally activated shape-memory behavior is governed not only by the nominal thermomechanical transition but also by the coupled balance among PDMS-mediated segmental mobility, switching monomer structure, network constraint, mechanical integrity, and elastic energy storage. However, quantitative thermomechanical shape-memory cycling in this study was focused on selected 15 wt% PDMS-MMA formulations. Future work will include systematic shape-memory testing over a broader PDMS-MMA composition range, optimization of programming and recovery conditions, and fabrication of more complex 4D-printable geometries to improve actuation controllability and practical applicability.

## Figures and Tables

**Table 1:** Chemical information and functional role of the resin components used for PDMS-MMA-modified photocurable networks.

Component	CAS No.	Molar Mass or *M_n_* (g/mol)	Transition-Related Information	Function in Formulation
TEGDMA	109-16-0	286.32	Contributes to glassy methacrylate network formation	Primary dimethacrylate monomer forming the glassy polymer backbone
TMPTMA	3290-92-4	338.4	Multifunctional crosslinker; *T_g_* not independently assigned	Crosslinker providing network constraint and elastic energy storage
tBA	1663-39-4	128.17	switching monomer; relatively flexible acrylate structure	Switching monomer for regulating thermomechanical transition
IBOA	5888-33-5	208.3	Bulky cyclic acrylate structure associated with restricted segmental mobility	Switching monomer affecting chain packing, stiffness, and thermomechanical transition
PDMS-MMA	58130-03-3	*M_n_* = 1300–1400	flexible siloxane segment	Soft segment for regulating segmental mobility
TPO	75980-60-8	348.38	Not applicable	Type-I photoinitiator for 405 nm UV curing
AUAO	Not disclosed by supplier/Proprietary	*M_n_* = 5200	Not independently assigned	Reactive aliphatic urethane acrylate oligomer for improving photocurable network formation, printability, and toughness

**Table 2:** Resin formulations of tBA- and IBOA-based PDMS-MMA-modified photocurable networks.

Resin	PDMS-MMA (wt%)	tBA (wt%)	IBOA (wt%)	TEGDMA (wt%)	Oligomer (wt%)	TMPTMA (wt%)	TPO (wt%)
T-0	0	20	0	58	10	10	2
T-5	5	20	0	53	10	10	2
T-10	10	20	0	48	10	10	2
T-15	15	20	0	43	10	10	2
I-0	0	0	20	58	10	10	2
I-5	5	0	20	53	10	10	2
I-10	10	0	20	48	10	10	2
I-15	15	0	20	43	10	10	2

**Table 3:** Degree of conversion (DC) of photocured tBA- and IBOA-based networks with varying PDMS-MMA content.

Specimen	PDMS-MMA (wt%)	DC (%)
T-0	0	92 ± 2
T-5	5	91 ± 2
T-10	10	90 ± 3
T-15	15	88 ± 3
I-0	0	91 ± 2
I-5	5	90 ± 2
I-10	10	89 ± 3
I-15	15	87 ± 3

**Table 4:** Characteristic thermal stability parameters obtained from TGA curves.

Specimen	T_5%_ (°C)	T_10%_ (°C)	Residual Mass at 500 °C (%)
T-0	297.0	335.1	0.8
T-05	289.2	333.3	1.0
T-10	293.3	329.8	1.4
T-15	292.1	329.8	1.3
I-0	299.5	341.1	0.4
I-05	291.29	327.7	0.6
I-10	294.9	330.6	0.8
I-15	293.3	329.0	0.9

**Table 5:** DMA-derived thermomechanical transition parameters of photocured tBA- and IBOA-based networks.

Specimen	*E″* Transition/Inflection Temperature (°C)	*E″* at Transition Point (MPa)	tan δ Peak Temperature, *T_g_*, DMA (°C)	tan δ Max
T-0	66.2	114.56	122.1	0.27
T-5	78.8	86.76	118.6	0.29
T-10	71.5	82.16	113.4	0.31
T-15	70.6	77.25	111.1	0.33
I-0	70.0	86.44	130.2	0.34
I-5	65.6	73.96	96.7	0.31
I-10	68.3	56.15	98.7	0.34
I-15	63.9	36.84	95.2	0.38

**Note:** Because the *E*″ curves exhibited broad relaxation behavior rather than sharply defined maxima, the *E*″ transition/inflection temperature was determined from the maximum decrease rate of the *E*″–temperature curve. The tan δ peak temperature was used as the representative DMA-derived thermomechanical transition temperature.

**Table 6:** Mechanical properties of photocured tBA- and IBOA-based networks with varying PDMS-MMA content at a fixed TMPTMA content of 10 wt% (25 ± 1 °C).

Specimen	PDMS-MMA (wt%)	Tensile Strength (MPa)	Young’s Modulus (MPa)
T-0	0	11.7	92.05
T-5	5	11.3	103.24
T-10	10	11.1	122.38
T-15	15	10.9	138.04
I-0	0	9.9	59.25
I-5	5	9.4	68.31
I-10	10	9.1	74.09
I-15	15	8.7	94.10

**Table 7:** Shape memory properties of representative PDMS-MMA-modified *T_g_*-related photocured networks.

Sample	Cycle	Shape Fixity (*R_f_*, %)	Shape Recovery (*R_r_*, %)	ε_cooling,load_ (%)	ε_fixed_ (%)	ε_rec_ (%)
I-15	1	95.32	91.51	2.556	2.437	0.207
	2	96.18	95.87	2.110	2.037	0.283
T-15	1	98.27	125.29	2.557	2.512	−0.635
	2	98.32	118.40	2.118	2.082	−1.135

**Table 8:** Comparison of the present PDMS-MMA-modified photocurable networks with representative vat-photopolymerization-based and PDMS/silicone-containing shape-memory polymer systems.

Material System	Processing Method	Key Design Feature	*R_f_* (%)	*R_r_* (%)	Remarks
This work, I-15	SLA-type vat photopolymerization	PDMS-MMA + IBOA + fixed TMPTMA network	95–96	92–96	Stable recovery
This work, T-15	SLA-type vat photopolymerization	PDMS-MMA + tBA + fixed TMPTMA network	98.3	118–125	Over-recovery attributed to residual stress release
tBA-co-DEGDA (tert-Butyl acrylate + di(ethylene glycol) diacrylate) [10]	SLA-type vat photopolymerization	tBA-co-DEGDA acrylate network based on dual-component phase switching	85–95.2	97–100	High durability over >20 cycles; complex 3D structures demonstrated
Methacrylate-based copolymers (BMA + PEGDMA/BPA/DEGDMA) [32]	PμSL (Projection microstereolithography)	BMA-based methacrylate copolymers with tunable crosslinker chemistry	>90	~95	Sequential shape recovery with large recoverable strain
PDMSUMA (Silicone urethane methacrylate) + PEGDMA/HEMA [26]	mSLA (Masked Stereolithography)	Synthesized PDMSUMA oligomers with PEGDMA/HEMA reactive diluent control	74.49%	100%	PDMS-containing resin; high recovery but composition-dependent fixity
Various vat-photopolymerization-based 4D printing materials [7]	SLA, DLP, PμSL, Volumetric	Review-based summary of vat-photopolymerizable 4D printing resin systems	92–99	95–110	Review-based comparison; performance depends on resin chemistry and protocol

**Note:** *R_f_* and *R_r_* denote the shape fixity ratio and shape recovery ratio, respectively. Direct quantitative comparison should be made with caution because the reported shape fixity and recovery ratios were obtained under different resin chemistries, programming temperatures, deformation modes, applied strain or stress levels, and cycle numbers.

## Data Availability

The original contributions presented in this study are included in the article. Further inquiries can be directed to the corresponding author.
